# Exploring the Double‐Edged Sword Effect of Responsible Leadership on Nurses’ Proactive Behavior: The Mediating Roles of Affective Commitment and Emotional Exhaustion

**DOI:** 10.1155/jonm/6642530

**Published:** 2026-06-25

**Authors:** Hang Zhang, Qinglin Wang, Junzhe Zhao, Minghui Wang, Jianjun Wang

**Affiliations:** ^1^ Department of Nursing, Huaihe Hospital of Henan University, Kaifeng, Henan, China, henu.edu.cn; ^2^ School of Psychology, Henan University, Kaifeng, Henan, China, henu.edu.cn; ^3^ School of Management, Jinan University, Guangzhou, Guangdong, China, jnu.edu.cn; ^4^ President’s Office, Huaihe Hospital of Henan University, Kaifeng, Henan, China, henu.edu.cn

**Keywords:** affective commitment, emotional exhaustion, proactive behavior, responsible leadership

## Abstract

**Background:**

While leadership has been identified as a critical antecedent, the mechanisms underlying the impact of responsible leadership, particularly its potential dual pathways, remain underexplored among nurses.

**Objectives:**

This study aims to investigate the mechanisms through which responsible leadership influences nurses’ proactive behavior, focusing on the mediating roles of affective commitment and emotional exhaustion.

**Methods:**

A multisource, multi‐time point survey was conducted. Data were collected from nurse–leader dyads across two time points, 45 days apart. A mediation model was constructed and tested using SPSS 24.0 and AMOS 19.0.

**Results:**

The empirical findings show that responsible leadership positively affects nurses’ proactive behavior (*b* = 0.280, SE = 0.118, *p* = 0.019 < 0.05). Both affective commitment (95% CI: [0.002, 0.109]) and emotional exhaustion (95% CI: [−0.112, −0.001]) of nurses serve as partial mediators in the relationship between responsible leadership and proactive behavior.

**Conclusions:**

Drawing on the Job Demands–Resources model, this study addresses the theoretical gap by revealing the paradoxical pathways through which a single leadership style can exert both motivational and health‐impairing effects, specifically in the case of responsible leadership. These findings suggest that nursing managers should foster responsible leadership to strengthen affective commitment and promote proactive behavior, while simultaneously mitigating the risk of emotional exhaustion through adequate resources, psychological safety, and regular support. This study provides empirical evidence to inform nursing management practices and workforce development strategies.

**Patient or Public Contribution:**

No patient or public contribution.

## 1. Introduction

In recent years, the nursing profession has grappled with high turnover rates and diminished well‐being [1]. Nurses experience elevated levels of anxiety and fear compared to their medical counterparts [2, 3], which not only reduces their proactive behavior but also undermines team stability [4, 5]. Therefore, enhancing the stability of nursing teams and fostering nurses’ motivation to take initiative have become urgent priorities in the healthcare field [6, 7].

Leadership has been identified as a crucial situational factor influencing nurses’ proactive behavior [8–10]. Employees closely observe and emulate their leaders, treating them as role models [11]. Given the inherent social responsibilities of nursing, nurses are expected to embrace societal duties alongside their professional roles, posing new challenges for head nurses who act as direct supervisors of nursing staff [12]. Maak and Pless [13] first introduced the concept of responsible leadership, defining it as “having responsibility for different stakeholders to achieve the common goals of the organization by building sustainable relationships, and in doing so, having the responsibility to reconcile conflicts and manage the interests of all parties”. Recent studies have explored its positive effects, such as enhancing affective commitment [14], organizational citizenship behavior [15, 16], and knowledge sharing [17].

However, scholarly attention to its negative behavioral outcomes remains limited [18]. Existing research primarily focuses on the buffering effects of responsible leadership against deviant behavior [19] and employees’ unethical behavior [20], while its potential adverse effects remain underexplored. Recent studies have revealed a “too‐much‐of‐a‐good‐thing” effect, wherein excessive responsible leadership may backfire and encourage unethical pro‐organizational behavior [21]. To address the incomplete understanding of leadership’s complex consequences, researchers have increasingly called for investigating the dual pathways through which a single leadership style influences employee outcomes [22]. For instance, beyond the predominant focus on the positive side of responsible leadership, Luo et al. [16] noted that its effects are not uniformly positive, depending on the psychological mechanisms activated. This aligns with broader leadership research suggesting that leaders can shape followers’ proactive behavior through both cognitive and affective mechanisms [23]. This raises a critical yet unresolved question regarding the complex mechanisms through which responsible leadership influences subordinate behavior. Accordingly, this study aims to investigate the double‐edged sword effect of responsible leadership on subordinates’ proactive behavior, thereby moving beyond a unidimensional perspective to reveal its more multifaceted influence.

### 1.1. Theoretical Framework

The Job Demands–Resources (JD‐R) model provides a valuable framework for understanding the dual nature of workplace experiences, positing that any work environment can be characterized by the dynamic interplay between job demands and job resources [24, 25]. Job resources refer to the physical, psychological, social, or organizational aspects of work that facilitate goal attainment, buffer the physiological and psychological costs of job demands, and foster personal growth and development [26]. Within this framework, leadership has been consistently identified as a critical social and interpersonal resource that shapes nurses’ well‐being and professional outcomes [27]. Conversely, job demands encompass the physical, social, or organizational aspects of work that require sustained physical or psychological effort, thereby being associated with certain physiological and psychological costs [24]. When job demands persistently outweigh available resources or exceed employees’ coping capacities, they can precipitate a state of depletion, manifesting as emotional exhaustion and diminished well‐being [24, 26, 28].

Drawing on the JD‐R model, leaders can serve as a crucial job resource by providing support and a sense of purpose, but they may also impose additional job demands through increased expectations and performance pressure [27]. This duality becomes especially pronounced in the context of responsible leadership. On the one hand, responsible leaders emphasize stakeholder engagement and ethical conduct. Through these behaviors, they offer valuable job resources that foster a supportive and meaningful work environment [13, 29]. On the other hand, their commitment to balancing diverse stakeholder interests and maintaining high ethical standards may inadvertently elevate job demands. Such pressures, if excessive, could lead to negative outcomes for their subordinates [21, 28].

According to the multilevel approach of the JD‐R model, nurses are not isolated individuals but are embedded within teams, which in turn form part of larger organizational structures [30]. This hierarchical embeddedness implies that nurses’ experiences of job demands and resources are shaped not only by individual‐level factors but also by team dynamics and organizational contexts. Within such a structure, leadership functions as a critical interpersonal and social resource that can influence team processes, shape collective perceptions of job demands, and ultimately affect individual well‐being and behavioral outcomes [27, 31].

### 1.2. Hypothesis Development

Affective commitment refers to employees’ emotional attachment to, identification with, and involvement in an organization [32]. According to the JD‐R model, leaders serve as a critical interpersonal resource by communicating with employees and providing the support necessary to perform their jobs [33, 34]. When leaders fulfill their obligations with a heightened sense of responsibility, employees exhibit higher levels of organizational commitment, including increased affective commitment [35]. Employees with strong affective commitment often feel an obligation to reciprocate toward the organization, engaging in extra‐role behaviors such as helping others [36]. Accordingly, we hypothesize the following:

 H1: Responsible leadership is positively associated with employee proactive behavior.
 H2: Affective commitment mediates the relationship between responsible leadership and employee proactive behavior.

Emotional exhaustion occurs when employees exert excessive effort at work, depleting their emotional resources and leading to feelings of fatigue and anxiety [37]. According to the JD‐R model, responsible leadership expands the scope of accountability and emphasizes the interests of diverse stakeholders. This expanded responsibility may therefore be perceived as a job demand that depletes employees’ limited resources [33, 34]. The broadened scope of responsibility increases employees’ workload and emotional strain, ultimately leading to emotional exhaustion. When employees experience emotional exhaustion, their engagement decreases, they become less inclined to assist colleagues, and they are less likely to offer suggestions that benefit the organization, resulting in a decline in work quality and proactive behavior [38–40]. Accordingly, we hypothesize the following:

 H3: Emotional exhaustion mediates the relationship between responsible leadership and proactive employee behavior.

The quality of the exchange relationship with leaders is a significant situational factor that should not be overlooked. A higher leader‐member exchange (LMX) relationship indicates that the responsible leaders foster a strong sense of community. In such a relationship, leaders not only provide necessary job resources but also show concern for employees’ family lives and emotional needs, enhancing their subordinates’ affective commitment. Furthermore, when responsible leaders demonstrate care for societal public interests and patient welfare, they serve as powerful role models for their team. This influence encourages subordinates to proactively address issues for the leader and derive satisfaction from these efforts, alongside their regular duties, which can help alleviate the feeling of emotional exhaustion [41]. Accordingly, we propose the following:

 H4: LMX positively moderates the mediating role of affective commitment between responsible leadership and proactive behavior. Affective commitment mediates more strongly when LMX is high and less strongly when it is low.
 H5: LMX negatively moderates the mediating role of emotional exhaustion between responsible leadership and proactive behavior. Emotional exhaustion mediates more strongly when LMX is low and less strongly when it is high.

Based on the proposed hypotheses, this study makes several contributions. First, by identifying affective commitment and emotional exhaustion as parallel mediators, it reveals the dual pathways through which responsible leadership influences nurses’ proactive behavior, offering a more balanced understanding of its effects. Second, drawing on the JD‐R model, it demonstrates how a single leadership style can simultaneously function as a job resource and a job demand, extending the application of the JD‐R model in healthcare contexts. Third, by examining the moderating role of LMX, this study tested LMX as a potential boundary condition for the effects of responsible leadership, but the moderating effects were not supported in this sample.

**FIGURE 1:**
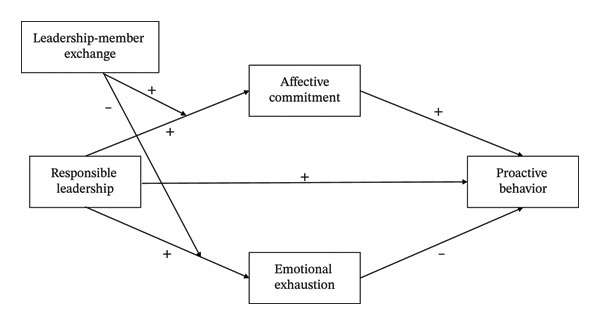
Diagram of the research model.

To better understand the relationships among responsible leadership, proactive behavior, affective commitment, emotional exhaustion, and LMX based on a hypothetical model (Figure 1).

## 2. Methods

### 2.1. Participants and Data Collection

This study recruited nurses and their corresponding head nurses from 47 departments across three comprehensive tertiary hospitals in central China. A convenience sampling method was employed to recruit participants. To enhance the representativeness of the sample, departments were selected across major clinical divisions, including internal medicine, surgery, obstetrics and gynecology, and pediatrics. The inclusion criteria for participants were as follows: (a) nurses held a valid registered nursing license; (b) head nurses had at least 1 year of leadership experience in their current role; and (c) all participants provided voluntary informed consent. Data were collected using a mixed‐mode survey administration: offline paper questionnaires were distributed to nurses during routine meetings, while online surveys (via a secure institutional platform) were administered to head nurses to accommodate their schedules.

This study employed a multisource, multi‐time point, and leader–employee pairing research paradigm. Data were collected at two time points, 45 days apart. Specifically, at Time Point 1, data on responsible leadership were collected. At Time Point 2, data on affective commitment, emotional exhaustion, proactive behavior, and LMX were gathered. Head nurses provided demographic information and evaluated nurses’ proactive behavior, while nurses provided demographic information and assessed responsible leadership, affective commitment, emotional exhaustion, and LMX.

The sample size was determined a priori using G∗Power 3.1, with an estimated minimum requirement of 176 participants to detect medium effect sizes (*f* ^2^ = 0.15) in a multiple regression model (*α* = 0.05, power = 0.95). To account for potential attrition, we oversampled by 120%, leading to an initial distribution of 400 nurse questionnaires and surveys for their corresponding head nurses (one per department). At Time Point 1, a total of 400 questionnaires were distributed to nurses and 400 to head nurses across the three hospitals. Of these, 338 matched pairs were retrieval, yielding a response rate of 84.50%. At Time Point 2, 338 questionnaires were again distributed to the nurses and head nurse who participated in Time Point 1, resulting in 226 matched pairs, with a response rate of 66.86%. These 226 matched questionnaires spanned 47 departments. Regarding educational attainment, 95.58% (216) of the nurses held a bachelor’s degree, and 4.42% (10) had a college degree. In terms of the tenure with the head nurse, 49.56% (112) of the nurses had worked together for less than five years, 32.30% (73) for six to 10 years, 14.60% (33) for 10 to 15 years, and 3.54% (8) for more than 15 years.

### 2.2. Responsible Leadership Scale

The responsible leadership scale developed by Voegtlin [42]. The scale consists of five items rated on a 5‐point Likert scale ranging from 1 (“strongly disagree”) to 5 (“strongly agree”), with higher scores indicating higher levels of responsible leadership. A sample item is: “My head nurse involves affected stakeholders in the decision‐making process”. The scale demonstrated satisfactory reliability with a Cronbach’s alpha of 0.949.

### 2.3. Proactive Behavior Scale

The proactive behavior scale developed by Griffin et al. [43] was employed. The scale consists of three dimensions: individual (three items), team (three items), and organizational (three items) and is scored on a 5‐point Likert scale ranging from 1 (“strongly disagree”) to 5 (“strongly agree”), with higher scores indicating higher levels of proactive behavior. A sample item is: “He/she will take the initiative to do his/her job in a better way”. The scale demonstrated satisfactory reliability with a Cronbach’s alpha of 0.971.

### 2.4. Affective Commitment Scale

The affective commitment dimension of Allen and Meyer’s [32] revised organizational commitment scale was employed. The scale consists of six items scored on a 5‐point Likert scale ranging from 1 (“strongly disagree”) to 5 (“strongly agree”), with higher scores indicating higher levels of affective commitment. A sample item is: “I have a strong sense of belonging to the organization I’m in now”. The scale demonstrated satisfactory reliability with a Cronbach’s alpha of 0.877.

### 2.5. Emotional Exhaustion Scale

The emotional exhaustion dimension of Maslach et al.’s [44] revised job burnout scale was employed. The scale consists of five items scored on a 5‐point Likert scale ranging from 1 (“strongly disagree”) to 5 (“strongly agree”), with higher scores indicating higher levels of emotional exhaustion. The scale was translated into Chinese following the version of Li and Shi [45]. A sample item is: “When I wake up in the morning and have to face the day’s work, I feel very tired.” The scale demonstrated satisfactory reliability with a Cronbach’s alpha of 0.872.

### 2.6. LMX Scale

The LMX scale developed by George and Uhl‐Bien [46] was employed. The scale consists of seven items scored on a 5‐point Likert scale ranging from 1 (“strongly disagree”) to 5 (“strongly agree”), with higher scores indicating higher levels of LMX. A sample item is: “I have full confidence in my head nurse and will defend his/her decisions even if he/she is not present.” The scale demonstrated satisfactory reliability with a Cronbach’s alpha of 0.911.

### 2.7. Control Variables

Demographic variables (nurses’ level of education, time spent working with the head nurse) were used as control variables in this study.

### 2.8. Data Analysis

Data were analyzed using SPSS 19.0 and AMOS 24.0. Preliminary analyses included descriptive statistics, correlation analyses, and assessment of common method bias using Harman’s single‐factor test. Prior to hypothesis testing, all measurement scales were scored by summing the item responses for each construct following the original scale developers’ recommendations. Specifically, responsible leadership, affective commitment, emotional exhaustion, LMX, and proactive behavior were each conceptualized as unidimensional constructs. Total scores were used as manifest variables for all subsequent analyses, as each construct was unidimensional with a moderate number of items. All scales were translated into Chinese following standard back‐translation procedures.

To assess the discriminant validity of the study constructs, confirmatory factor analysis (CFA) was conducted using AMOS 24.0. Model fit was evaluated using multiple indices, including the chi‐square statistic (*χ* ^2^), degrees of freedom (d*f*), the ratio of chi‐square to degrees of freedom (*χ* ^2^/d*f*), comparative fit index (CFI), Tucker–Lewis index (TLI), root mean square error of approximation (RMSEA), and standardized root mean square residual (SRMR). Acceptable model fit was defined as *χ* ^2^/d*f* < 3, CFI and TLI ≥ 0.90, RMSEA ≤ 0.08, and SRMR ≤ 0.08 [47]. In addition, convergent validity was assessed using composite reliability (CR) and average variance extracted (AVE), with CR > 0.70 and AVE > 0.50 indicating adequate convergent validity [48].

To test the proposed hypotheses, we employed Hayes’ PROCESS macro for SPSS (Model 4 for simple mediation and Model 7 for moderated mediation; [49]). PROCESS Model 4 was used to test the parallel mediation hypotheses (H2 and H3), examining the indirect effects of responsible leadership on proactive behavior through affective commitment and emotional exhaustion simultaneously. This model is theoretically appropriate as it allows the independent variable to influence the dependent variable through two distinct mediators operating in parallel. PROCESS Model 7 was used to test the moderated mediation hypotheses (H4 and H5), examining whether LMX moderated the indirect effects. This model aligns with the theoretical framework by specifying that the moderator influences the first stage of the mediation pathways (the relationship between responsible leadership and each mediator), while leaving the remaining paths unmoderated. All analyses controlled for nurses’ level of education and time spent working with the head nurse as potential covariates. Indirect effects were estimated using bias‐corrected bootstrapping with 5000 resamples, and 95% confidence intervals were computed. An indirect effect was considered statistically significant if the confidence interval did not contain zero.

### 2.9. Ethical Considerations

This study was conducted in accordance with the Declaration of Helsinki and approved by the Ethics Committee of Henan University (No. 20230316002). Prior to participation, all nurses were informed of the purpose of the study, the voluntary nature of their participation, and their right to withdraw at any time without consequence. Participants were assured that all responses would be treated as strictly confidential and used solely for scientific research purposes. To protect participant privacy, data were anonymized prior to analysis, and no identifying information was collected from either nurses or their head nurses.

## 3. Results

### 3.1. Common Method Bias Test

In this study, common method bias was mitigated through the use of two time points and multiple subject sources of data. The Harman’s single‐factor test was employed to assess common‐method bias [50]. The results indicated two factors with eigenvalues greater than 1. The first factor explained 37.92% of the variance, which is less than 40%, implying that there was no serious common method bias in this study.

### 3.2. CFA

Prior to hypothesis testing, a structural equation model was constructed with the five latent variables of responsible leadership, proactive behavior, affective commitment, emotional exhaustion, and LMX. As shown in Table 1, the fit of the five‐factor model was significantly superior to that of alternative models, indicating a high level of discriminant validity among the five variables in this study.

**TABLE 1:** Confirmatory factor analysis test.

Model	*χ* ^2^	d*f*	*χ* ^2^/d*f*	RMSEA	CFI	TLI	SRMR
Five factors model: 1, 2, 3, 4, 5	872.963	450	1.940	0.064	0.936	0.929	0.069
Four factors model: 1, 2, 3 + 4, 5	1237.584	454	2.726	0.087	0.881	0.870	0.090
Three factors model: 1, 2 + 3 + 4, 5	2170.393	459	4.729	0.127	0.654	0.627	0.161
Two factors model: 1, 2 + 3+4 + 5	2736.814	460	5.950	0.147	0.731	0.707	0.187
One factor model: 1 + 2+3 + 4+5	3879.604	461	8.416	0.180	0.481	0.442	0.215

*Note:* 1 = responsible leadership, 2 = proactive behavior, 3 = affective commitment, 4 = emotional exhaustion, 5 = leader‐member exchange. *χ* ^2^ = chi‐square; the five‐factor model was compared with alternative nested models. Acceptable model fit was defined as *χ* ^2^ */*d*f* < 3, CFI and TLI ≥ 0.90, RMSEA ≤ 0.08, and SRMR ≤ 0.08.

Abbreviations: CFI, comparative fit index; d*f*, degrees of freedom; RMSEA, root mean square error of approximation; SRMR, standardized root mean square residual; TLI, Tucker–Lewis index.

To further assess convergent validity, CR and AVE were calculated. As presented in Table 2, the CR values for the five constructs ranged from 0.858 to 0.970 (≥ 0.70). The AVE values ranged from 0.549 to 0.797 (≥ 0.50). These results indicate adequate internal consistency and convergent validity [48].

**TABLE 2:** Convergent validity.

Variable	CR	AVE
Responsible leadership	0.951	0.797
Affective commitment	0.885	0.567
Emotional exhaustion	0.858	0.549
Leader‐member exchange	0.915	0.606
Proactive behavior	0.970	0.783

*Note:* Acceptable convergent validity was defined as CR > 0.70 and AVE > 0.50. All constructs exceeded these thresholds, indicating adequate convergent validity.

Abbreviations: AVE, average variance extracted; CR, composite reliability.

### 3.3. Descriptive Statistics and Correlation Analysis

As presented in Table 3, responsible leadership at Time Point 1 is significantly and positively correlated with affective commitment (*r* = 0.185, *p* < 0.01) and emotional exhaustion (*r* = 0.162, *p* < 0.05) at Time Point 2. Additionally, there is a significant positive correlation between affective commitment and proactive behavior (*r* = 0.215, *p* < 0.01) at Time Point 2 and a significant negative correlation between emotional exhaustion and proactive behavior (*r* = −0.207, *p* < 0.01) at Time Point 2.

**TABLE 3:** Descriptive statistics and correlation coefficients (*n* = 226).

Variable	*M*	SD	1	2	3	4	5	6	7
1. Education level of nurses	2.957	0.204							
2. Time spent together	1.727	0.839	−0.021						
3. Responsible leadership	19.703	3.299	−0.013	−0.029					
4. Leader‐member exchange	27.573	3.897	−0.007	0.019	0.162^∗^				
5. Affective commitment	23.225	3.439	−0.025	−0.005	0.185^∗∗^	0.452^∗∗^			
6. Emotional exhaustion	14.826	3.007	−0.136^∗^	0.076	0.162^∗^	−0.207^∗∗^	−0.390^∗∗^		
7. Proactive behavior	38.013	5.896	0.165^∗^	0.036	0.143^∗^	0.197^∗∗^	0.215^∗∗^	−0.207^∗∗^	

*Note:* ^∗^ means *p* < 0.05; ^∗∗^ means *p* < 0.01. *M* = mean; *n* = number of participants. All variables were measured using summated scores.

Abbreviation: SD, standard deviation.

### 3.4. Mediational Analyses

Hierarchical regression was employed to test the mediating effect among the variables. The results indicated that responsible leadership significantly and positively influenced nurses’ proactive behaviors (*b* = 0.280, SE = 0.118, *t* = 2.370, *p* = 0.019 < 0.05). Responsible leadership also significantly and positively affected nurses’ affective commitment (*b* = 0.180, SE = 0.068, *t* = 2.631, *p* = 0.009 < 0.01) and emotional exhaustion (*b* = 0.146, SE = 0.059, *t* = 2.475, *p* = 0.014 < 0.05), affective commitment significantly and positively influenced nurses’ proactive behaviors (*b* = 0.256, SE = 0.124, *t* = 2.075, *p* = 0.039 < 0.05), while emotional exhaustion significantly and negatively affected proactive behavior (*b* = −0.338, SE = 0.143, *t* = −2.360, *p* = 0.019 < 0.05). The mediating effects were further examined using the bootstrap method, as shown in Table 4. Responsible leadership influences nurses’ proactive behaviors through affective commitment (95% CI [0.002, 0.109]) and through emotional exhaustion (95% CI [−0.112, −0.001]). Hypotheses 1, 2, and 3 were validated, demonstrating that affective commitment positively mediates the relationship between responsible leadership and proactive behavior, while emotional exhaustion negatively mediates this relationship.

**TABLE 4:** Indirect effect values of each path (*n* = 226).

	Effect	Confidence interval (95%)		Boot SE	Relative indirect effect value (%)
		LL 95%	UL 95%		
*X* ⟶ M1 ⟶ *Y*	0.046	0.002	0.109	0.028	16.670
*X* ⟶ M2 ⟶ *Y*	−0.049	−0.112	−0.001	0.029	17.830
Total indirect effect	−0.003	−0.093	0.092	0.047	1.157

*Note: X* represents responsible leadership, Y represents proactive behavior, M1 represents affective commitment, M2 represents emotional exhaustion. Effect = unstandardized indirect effect; Boot SE = bootstrapped standard error; confidence interval (95%) = bias‐corrected bootstrap confidence interval based on 5000 resamples. An indirect effect was considered statistically significant if the 95% confidence interval did not contain zero. Relative indirect effect value represents the proportion of the total effect accounted for by each mediator.

### 3.5. Moderated Mediation Analyses

**FIGURE 2:**
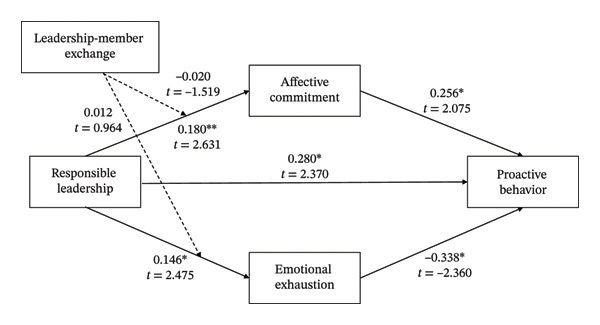
Research model results. *Note:* ^∗^ means *p* < 0.05, ^∗∗^ means *p* < 0.01. Solid lines represent significant paths; dashed lines represent nonsignificant paths, *t*‐values are presented below each path coefficient.

The test for moderating effects revealed that the interaction between responsible leadership and LMX did not significantly influence nurses’ affective commitment (*b* = −0.020, SE = 0.013, *t* = −1.519, *p* = 0.130 > 0.05), thus not supporting hypothesis 4. Similarly, the interaction between responsible leadership and LMX did not significantly affect nurses’ emotional exhaustion (*b* = 0.012, SE = 0.012, *t* = −0.964, *p* = 0.336 > 0.05), thereby not supporting hypothesis 5. The results of the research model are illustrated in Figure 2.

Based on the analyses above, PROCESS Model 7 was employed to test the moderated mediation model. The results indicated that when affective commitment was the mediating variable, the moderated mediation effect was −0.005 (95% CI [−0.016, 0.001]). When emotional exhaustion was the mediating variable, the moderated mediation effect was −0.004 (95% CI [−0.017, 0.004]). Contrary to H4 and H5, the indirect effects of responsible leadership on proactive behavior through affective commitment and emotional exhaustion were not moderated by LMX. The conditional indirect effects were non‐significant across different levels of LMX, suggesting that the mediating mechanisms may be relatively stable and less dependent on leader‐subordinate relationship quality.

## 4. Discussion

This study enhances the understanding of the mechanism through which responsible leadership influence on proactive behaviors, particularly from an affective perspective. While prior research has predominantly emphasized the positive outcomes of responsible leadership—such as enhanced affective commitment and organizational citizenship behavior—the potential costs embedded in this leadership approach have received considerably less attention [18]. Moreover, existing studies have largely examined these effects in isolation, overlooking the possibility that responsible leadership may activate dual, opposing psychological pathways. Based on the JD‐R model, this study proposes a dual‐pathway framework in which responsible leadership enhances proactive behavior through affective commitment (a motivational process) while diminishing it through emotional exhaustion (a health‐impairment process). By identifying these parallel mediating mechanisms, this study fills a significant gap in the literature and provides a more nuanced understanding of how responsible leadership shapes nurses’ behavioral outcomes.

### 4.1. Hypothesis Testing: The Dual Pathways of Responsible Leadership

Hypothesis 1 proposed a direct positive relationship between responsible leadership and proactive behavior. Our results supported this hypothesis, showing that responsible leadership significantly and positively influenced nurses’ proactive behavior. This finding aligns with prior research demonstrating that responsible leadership, through its emphasis on stakeholder engagement and ethical conduct, can directly motivate employees to engage in discretionary, future‐oriented actions [15, 16]. From the perspective of the JD‐R model, responsible leadership can be understood as a critical job resource that provides employees with support, guidance, and a sense of purpose. These resources enhance employees’ intrinsic motivation and willingness to invest effort beyond formal job requirements, thereby directly promoting proactive behavior [25, 26].

According to the JD‐R model, Hypotheses 2 and 3 proposed parallel mediating roles of affective commitment and emotional exhaustion. Our results supported both hypotheses. Consistent with Hypothesis 2, responsible leadership enhanced proactive behavior by increasing nurses’ affective commitment. Value‐based leadership styles foster emotional attachment to the organization, which in turn motivates employees to engage in extra‐role behaviors [14, 36]. When responsible leaders demonstrate genuine concern for stakeholders and ethical conduct, nurses internalize these values, developing a stronger sense of identification and belonging that translates into proactive actions.

Consistent with Hypothesis 3, responsible leadership also indirectly reduced proactive behavior by contributing to emotional exhaustion. This finding extends previous research on the potential costs of stakeholder‐oriented leadership [21, 28]. The expanded scope of accountability inherent in responsible leadership (including heightened expectations to balance diverse stakeholder interests) can be perceived as a job demand that depletes nurses’ emotional resources, leading to fatigue and reduced engagement. When nurses experience emotional exhaustion, they become less inclined to invest discretionary effort, thereby diminishing their proactive behaviors. Taken together, the paradoxical nature of responsible leadership exerts a direct positive effect on proactive behavior while also operating through opposing indirect pathways: one motivational (affective commitment) and one health‐impairing (emotional exhaustion). This dual‐pathway finding challenges overly optimistic views of positive leadership styles and underscores the importance of considering both the benefits and costs when evaluating leadership effectiveness.

In contrast, the moderated mediation hypotheses (H4 and H5) were not supported. Our results showed that LMX did not significantly moderate the indirect effects of responsible leadership on proactive behavior through either affective commitment or emotional exhaustion. This nonsignificant finding may reflect a substitution effect between different types of job resources. LMX is commonly understood as a social resource that buffers job demands and motivates employees [25, 26]. Yet responsible leadership itself embodies a broad set of resources, including relational transparency, ethical conduct, and equitable treatment [13]. When responsible leadership is characterized by such behaviors, the added value of LMX may be limited in shaping employees’ affective and emotional responses. Moreover, responsible leaders tend to emphasize fairness and stakeholder welfare, potentially reducing the in‐group bias often associated with high‐quality LMX relationships. As a result, the typical disparities in resource allocation linked to LMX may be weakened, making its moderating role less pronounced. This pattern aligns with the JD‐R model’s view that the impact of any given job resource depends on the broader resource context; when resources are already abundant, the marginal contribution of an additional resource may be limited [25, 30].

### 4.2. Theoretical and Practical Implications

This study offers theoretical contributions. First, it reveals how responsible leadership influences nurses’ proactive behavior through parallel mediating pathways (affective commitment and emotional exhaustion). This dual‐pathway framework provides a more complete understanding of how responsible leadership shapes employee outcomes in nursing contexts. In addition, this study extends the JD‐R model by demonstrating that a single leadership style can simultaneously function as a job resource (enhancing affective commitment) and a job demand (inducing emotional exhaustion). This parallel mediation enriches the JD‐R model by showing that resources and demands can originate from the same source. Second, these findings challenge overly optimistic views of positive leadership styles. While prior research has largely emphasized the benefits of responsible leadership [14, 15], our results show that its effects are not uniformly positive. By revealing this double‐edged sword effect, this study underscores the importance of considering both the benefits and costs when evaluating leadership outcomes.

This study also offers actionable insights for nursing managers. First, to strengthen the positive pathway through affective commitment, managers should implement concrete strategies to build nurses’ emotional attachment to the organization: (1) provide consistent recognition for individual contributions, (2) foster a sense of belonging through inclusive team practices, (3) offer emotional support during challenging work periods, and (4) align daily management actions with nurses’ personal and professional values. Second, to mitigate the negative pathway through emotional exhaustion, managers must adopt distinct demand‐oriented strategies: (1) actively monitor workload intensity, (2) ensure adequate staffing levels to prevent chronic overload, (3) guarantee sufficient recovery time between shifts, (4) implement flexible scheduling to accommodate work‐life balance, and (5) carefully calibrate role demands to avoid unrealistic expectations. Additionally, leadership training should be emphasized to help nurse managers balance these dual responsibilities: providing resources to foster commitment while controlling demands to prevent exhaustion.

### 4.3. Limitations and Future Research

Several limitations should be acknowledged. First, the study was conducted in tertiary hospitals in central China, a context characterized by high patient‐to‐nurse ratios, rigid hierarchical structures, and strong collectivist cultural values [12], which may limit generalizability to other healthcare systems or cultural settings. Second, although the multisource and multi‐time point design helps mitigate common method bias, key constructs were assessed using self‐report measures, which may introduce response bias and fail to capture objective leadership behaviors or physiological markers of exhaustion. Third, the absence of qualitative data limits understanding of how nurses experience the dual effects of responsible leadership in their daily work.

Building on these findings, several avenues for future research emerge. First, to capture the lived experiences of nurses and deepen understanding of the underlying mechanisms, future studies could employ qualitative methods such as in‐depth interviews or diary studies, alongside leader–employee dyadic designs to reduce common method bias. Second, to enhance generalizability, cross‐cultural and cross‐regional comparisons involving diverse healthcare systems and organizational contexts are warranted. Third, the non‐significant moderating effects of LMX offer important opportunities to explore alternative boundary conditions, such as organizational ethical climate, perceived organizational support, psychological safety, or cultural values like power distance and collectivism. By pursuing these avenues, future research can further unravel the complex mechanisms through which responsible leadership shapes nurse well‐being and performance.

## 5. Conclusion

This study explored the relationship between responsible leadership and nurses’ proactive behaviors in tertiary hospitals in central China using a leader–employee dyadic, multi‐time point design. The findings reveal that responsible leadership exerts a dual‐path influence on nurses’ proactive behaviors: it promotes proactive behaviors by enhancing affective commitment while simultaneously inhibiting them by increasing emotional exhaustion. By identifying these parallel mediating pathways, this study enriches the JD‐R model by demonstrating how a single leadership style can function as both a resource and a demand. Practically, nursing managers should foster affective commitment through emotional support and resource provision while mitigating emotional exhaustion by monitoring task demands. Overall, this study opens new avenues for examining the unintended consequences of positive leadership styles, contributing to a more balanced perspective in both leadership research and healthcare management practice.

## Author Contributions

Conceptualization, Hang Zhang, Qinglin Wang, Junzhe Zhao, and Minghui Wang; methodology, Hang Zhang and Qinglin Wang; writing–original draft preparation, Hang Zhang and Qinglin Wang; writing–review and editing, Hang Zhang, Qinglin Wang, and Junzhe Zhao; visualization, Hang Zhang, Qinglin Wang, Junzhe Zhao, and Minghui Wang; writing–review and editing, Jianjun Wang.

## Funding

This work was supported by the National Social Science Fund of China (award number: 24AGL037).

## Disclosure

All authors have read and agreed to the published version of the manuscript.

## Ethics Statement

This study was conducted in accordance with the Declaration of Helsinki and approved by the Ethics Committee of Henan University (No. 20230316002).

## Consent

Informed consent was obtained from all subjects involved in the study.

## Conflicts of Interest

The authors declare no conflicts of interest.

## Data Availability

The data presented in this study are available on request from the corresponding author. The data are not publicly available due to ethical restrictions.

## References

[1] Zhao Y. , Wang H. , Sun D. et al., Job Satisfaction, Resilience and Social Support in Relation to Nurses’ Turnover Intention Based on the Theory of Planned Behaviour: A Structural Equation Modelling Approach, International Journal of Nursing Practice. (2021) 7, no. 6, 10.1111/ijn.12941. 33856093

[2] Yuan H. , Luo L. , Wu J. Y. , Hu D. , and Lei K. , Mental Health Status and Coping Strategies Among Medical Staff During Outbreak of Coronavirus Disease 2019, Medical Journal of Wuhan University. (2020) 41, no. 6, 883–888, 10.14188/j.1671-8852.2020.0229.

[3] Yu M. and Lee H. , Impact of Resilience and Job Involvement on Turnover Intention of New Graduate Nurses Using Structural Equation Modeling: Turnover Intention of New Nurses, Japan Journal of Nursing Science. (2018) 15, no. 4, 351–362, 10.1111/jjns.12210. 29508523

[4] Yu Q. , Jiang L. L. , and Yin D. , Study on Correlation Between Professional Well-Being, Professional Mission and Professional Commitment in Outpatient Nurses, Occupation and Health. (2023) 39, no. 3, 375–379, 10.2147/PRBM.S446448.

[5] Rainbow J. G. and Steege L. M. , Presenteeism in Nursing: an Evolutionary Concept Analysis, Nursing Outlook. (2017) 65, no. 5, 615–623, 10.1016/j.outlook.2017.03.005. 28416202

[6] Hou N. N. , Yuan J. H. , Luan S. W. , Wang Y. , and Wang W. N. , A Study of the Correlation Between Young Clinical Nurses’ Career Adaptability and Future Time Insight and Mental Climate of the Work Environment, Chinese General Practice Nursing. (2023) 21, no. 6, 732–736.

[7] Awad N. H. A. , Zabady H. A. H. , Elbialy G. G. , and Ashour H. M. A. A. , Entrepreneurial Leadership, Nurses’ Proactive Work Behavior, and Career Adaptability: A Structural Equation Model, BMC Nursing. (2024) 23, no. 1, 10.1186/s12912-024-01804-4. PMC10900744 38413924

[8] Hobfoll S. E. , Conservation of Resources: A New Attempt at Conceptualizing Stress, American Psychologist. (1989) 44, no. 3, 513–524, 10.1037//0003-066x.44.3.513. 2648906

[9] Wang C. J. and Yang I. H. , Why and How Does Empowering Leadership Promote Proactive Work Behavior? An Examination With a Serial Mediation Model Among Hotel Employees, International Journal of Environmental Research and Public Health. (2021) 18, no. 5, 10.3390/ijerph18052386. PMC7967770 33804442

[10] Brown M. E. , Trevino L. K. , and Harrison D. A. , Ethical Leadership: A Social Learning Perspective for Construct Development and Testing, Organizational Behavior and Human Decision Processes. (2005) 97, no. 2, 17–134, 10.1016/j.obhdp.2005.03.002.

[11] Lord R. G. and Brown D. J. , Leadership, Values, and Subordinate Self-Concepts, The Leadership Quarterly. (2001) 12, no. 2, 133–152, 10.1016/S1048-9843(01)00072-8.

[12] Zeng L. , Liu G. , Feng F. et al., Effects of Compassion Satisfaction and Compassion Fatigue on Posttraumatic Growth of Psychiatric Nurses: A Cross-Sectional Study, International Journal of Nursing Practice. (2024) 30, no. 4, 10.1111/ijn.13215. 37968111

[13] Maak T. and Pless N. M. , Responsible Leadership in a Stakeholder Society: A Relational Perspective, Journal of Business Ethics. (2006) 66, no. 1, 99–115, 10.1007/s10551-006-9047-z.

[14] Gomes J. F. S. , Marques T. , and Cabral C. , Responsible Leadership, Organizational Commitment, and Work Engagement: The Mediator Role of Organizational Identification, Nonprofit Management and Leadership. (2022) 33, no. 1, 89–108, 10.1002/nml.21502.

[15] Freire C. and Gonçalves J. , The Relationship Between Responsible Leadership and Organizational Citizenship Behavior in the Hospitality Industry, Sustainability. (2021) 13, no. 9, 10.3390/su13094705.

[16] Luo J. , Ng L. P. , and Choong Y. O. , The Effect of Responsible Leadership on Organizational Citizenship Behavior: Double Mediation of Gratitude and Organizational Identification, BMC Psychology. (2025) 13, no. 1, 10.1186/s40359-024-02337-w. PMC11699642 39754288

[17] Haider S. A. , Akbar A. , Tehseen S. , Pouvola P. , and Jaleel F. , The Impact of Responsible Leadership on Knowledge Sharing Behavior Through the Mediating Role of Person-Organization Fit and Moderating Role of Higher Educational Institute Culture, Journal of Innovation & Knowledge. (2022) 7, no. 4, 10.1016/j.jik.2022.100265.

[18] Javed M. , Pless N. , Waldman D. A. et al., What, When, and How of Responsible Leadership: Taking Stock of Eighteen Years of Research and a Future Agenda, Journal of Management Studies. (2025) 62, no. 7, 3182–3219, 10.1111/joms.13157.

[19] Ahmad M. S. , Iqbal F. , Siddique R. , Abbas S. , and Fakhr Z. , Responsible Leadership and Workplace Deviant Behaviour: Modelling Trust and Turnover Intention as Mediator, The Leadership & Organization Development Journal. (2020) 41, no. 7, 939–952, 10.1108/lodj-05-2019-0212.

[20] Cheng K. , Wei F. , and Lin Y. , The Trickle-Down Effect of Responsible Leadership on Unethical Pro-Organizational Behavior: The Moderating Role of Leader-Follower Value Congruence, Journal of Business Research. (2019) 102, 34–43, 10.1016/j.jbusres.2019.04.044.

[21] Wang Z. , Wang Y. , and He Q. , Too Much of a Good Thing? The Curvilinear Effect of Responsible Leadership on Unethical Pro-Organizational Behavior in Projects, Project Management Journal. (2025) 56, no. 3, 1–23, 10.1177/87569728251403053.

[22] Heim I. , Laker B. , and Tabaeifard S. J. , Responsible Leadership: A Systematic Literature Review, Theoretical Framework, and Future Research Directions, Journal of Business Research. (2026) 203, 10.1016/j.jbusres.2025.115801.

[23] Zhang D. , Wang X. , and Zhang S. , The Influence of Shared Leadership on Taking Charge Behavior: Dual Perspective of Cognition–Affection, Journal of Management and Organization. (2024) 30, no. 6, 2105–2125, 10.1017/jmo.2023.70.

[24] Demerouti E. , Bakker A. B. , Nachreiner F. , and Schaufeli W. B. , The Job Demand Resources Model of Burnout, Journal of Applied Psychology. (2001) 86, no. 3, 499–512, 10.1037/0021-9010.86.3.499. 11419809

[25] Bakker A. B. and Demerouti E. , The Job Demands-Resources Model: State of the Art, Journal of Managerial Psychology. (2007) 22, no. 3, 309–328, 10.1108/02683940710733115.

[26] Schaufeli W. B. and Bakker A. B. , Job Demands, Job Resources, and Their Relationship With Burnout and Engagement: A Multi-Sample Study, Journal of Organizational Behavior. (2004) 25, no. 3, 293–315, 10.1002/job.248.

[27] Montenegro Mendez S. , Laguia Gonzalez A. , and Moriano Leon J. A. , Is Leadership a Resource? A Systematic Review of Its Role in Burnout and Engagement Among Nurses Within the JD-R Model, Journal of Nursing Management. (2025) 2025, 8853148–27, 10.1155/jonm/8853148. 41355851 PMC12677997

[28] Czakert J. P. , Leiva Ureña D. , and Berger R. G. , How Transformational Leadership Affects the Off-Work Recovery of Daily Personal Energy Resources via Work Engagement: Resource and Demand-Based Pathways, Spanish Journal of Psychology. (2024) 27, no. 11, 10.1017/SJP.2024.12. 38575505

[29] Voegtlin C. , Patzer M. , and Scherer A. G. , Responsible Leadership in Global Business: A New Approach to Leadership and Its Multi-Level Outcomes, Journal of Business Ethics. (2012) 105, no. 1, 1–16, 10.1007/s10551-011-0952-4.

[30] Bakker A. B. , Demerouti E. , Sanz-Vergel A. , and Rodriguez-Munoz A. , La Teoría de las Demandas y Recursos Laborales: Nuevos Desarrollos en la Última Década, Journal of Work and Organizational Psychology. (2023) 39, no. 3, 157–167, 10.5093/jwop2023a17.

[31] Albrecht S. L. , Bakker A. B. , Gruman J. A. , Macey W. H. , and Saks A. M. , Employee Engagement, Human Resource Management Practices and Competitive Advantage: An Integrated Approach, Journal of Organizational Effectiveness. People and Performance. (2015) 2, no. 1, 7–35, 10.1108/JOEPP-08-2014-0042.

[32] Allen N. J. and Meyer J. P. , A Three-Component Conceptualization of Organizational Commitment, Human Resource Management Review. (1991) 1, no. 1, 61–89, 10.1016/1053-4822(91)90011-Z.

[33] Demerouti E. and Bakker A. B. , Job Demands-Resources Theory in Times of Crises: New Propositions, Organizational Psychology Review. (2023) 13, no. 3, 209–236, 10.1177/20413866221135022.

[34] Bennett J. , Tepper N. D. , Lisa S. L. et al., Examining Follower Responses to Transformational Leadership From a Dynamic, Person-Environment Fit Perspective, Academy of Management Journal. (2014) 61, no. 4, 1343–1368, 10.5465/amj.2014.0163.

[35] Amlan H. , Mario F. , and Peter C. , The Relationship Between Responsible Leadership and Organizational Commitment and the Mediating Effect of Employee Turnover Intentions: An Empirical Study With Australian Employees, Journal of Business Ethics. (2019) 156, no. 3, 759–774, 10.1007/s10551-017-3575-6.

[36] Kang W. Z. , Guo L. Y. , Zhang J. C. , and Zhang J. C. , The Influence of Authoritarian Leadership on Employee Helping Behavior: The Chain Mediating Role of Perceived Insider Status and Affective Commitment, Journal of Technology Economics. (2019) 38, no. 11, 57–63+82.

[37] Guo Y. X. and Su Y. , Conceptual Structure and Scale of Responsible Leadership, Journal of Technology Economics. (2017) 36, no. 10, 77–83.

[38] St-Jean É. , Tremblay M. , Chouchane R. , and Saunders C. , Career Shock and the Impact of Stress, Emotional Exhaustion, and Resources on Entrepreneurial Career Commitment During the COVID-19 Pandemic, International Journal of Entrepreneurial Behavior & Research. (2023) 29, no. 8, 1927–1949, 10.1108/IJEBR-03-2022-0280.

[39] Lin S. H. and Johnson R. E. , A Suggestion to Improve a Day Keeps Your Depletion Away: Examining Promotive and Prohibitive Voice Behaviors Within a Regulatory Focus and Ego Depletion Framework, Journal of Applied Psychology. (2015) 100, no. 5, 1381–1397, 10.1037/apl0000018. 25706447

[40] John P. T. , Daniel J. B. , Bonnie H. C. , Ivona H. , and David Z. , Too Drained to Help: A Resource Depletion Perspective on Daily Interpersonal Citizenship Behaviors, Journal of Applied Psychology. (2015) 100, no. 1, 227–236, 10.1037/a0038082. 25314365

[41] Zhang P. C. , Jiang M. Q. , Li J. , and Chen F. , An Investigation on the “Double Edged Sward” Effect of Leader’s Power Sharing on Individual Idea Championing, Journal of Management Science. (2018) 29, no. 3, 40–50.

[42] Voegtlin C. , Development of a Scale Measuring Discursive Responsible Leadership, Journal of Business Ethics. (2011) 98, no. 1, 57–73, 10.1007/s10551-011-1020-9.

[43] Griffin M. A. , Neal A. , and Parker S. K. , A New Model of Work Role Performance: Positive Behavior in Uncertain and Interdependent Contexts, Academy of Management Journal. (2007) 50, no. 2, 327–347, 10.5465/AMJ.2007.24634438.

[44] Maslach C. , Jackson S. E. , and Leiter M. P. , MBI: Maslach Burnout Inventory-General Surveys, 1996, Consulting Psychologists Press.

[45] Li C. P. and Shi K. , The Influence of Distributive Justice and Procedural Justice on Job Burnout, Acta Psychology Sinica. (2003) 35, no. 5, 677–684.

[46] George B. G. and Uhl-Bien M. , Relationship-Based Approach to Leadership: Development of Leader-Member Exchange (LMX) Theory of Leadership Over 25 years: Applying a Multi-Level Multi-Domain Perspective, The Leadership Quarterly. (1995) 6, no. 2, 219–247, 10.1016/1048-9843(95)90036-5.

[47] Hu L. T. and Bentler P. M. , Cutoff Criteria for Fit Indexes in Covariance Structure Analysis: Conventional Criteria Versus New Alternatives, Structural Equation Modeling. (1999) 6, no. 1, 1–55, 10.1080/10705519909540118.

[48] Fornell C. and Larcker D. F. , Evaluating Structural Equation Models With Unobservable Variables and Measurement Error, Journal of Marketing Research. (1981) 18, no. 1, 39–50, 10.2307/3151312.

[49] Hayes A. F. , Introduction to Mediation, Moderation, and Conditional Process Analysis: A Regression-Based Approach, 2013, Guilford Press.

[50] Zhou H. and Long L. R. , Statistical Remedies for Common Method Biases, Advances in Psychological Science. (2004) 22, no. 6, 942–950.

